# Metabolic and transcriptomic changes induced in host during hypersensitive response mediated resistance in rice against the Asian rice gall midge

**DOI:** 10.1186/s12284-016-0077-6

**Published:** 2016-02-19

**Authors:** Ruchi Agarrwal, Ayyagari Phani Padmakumari, Jagadish S. Bentur, Suresh Nair

**Affiliations:** International Centre for Genetic Engineering and Biotechnology (ICGEB), Aruna Asaf Ali Marg, New Delhi, 110067 India; Indian Institute of Rice Research (formerly Directorate of Rice Research), Rajendranagar, Hyderabad, 500030 India; Present address: AgriBiotech Foundation, Rajendranagar, Hyderabad, 500030 India

**Keywords:** Aminotransferase, GABA, Hypersensitive response, Insect-plant interaction, Rice-gall midge interaction, Metabolic profiling, Singlet oxygen, GC-MS, Microarray, Transcript profiling

## Abstract

**Background:**

An incompatible interaction between rice (*Oryza sativa*) and the Asian rice gall midge (AGM, *Orseolia oryzae* Wood-Mason), that is usually manifested through a hypersensitive response (HR), represents an intricate relationship between the resistant host and its avirulent pest. We investigated changes in the transcriptome and metabolome of the host (*indica* rice variety: RP2068-18-3-5, RP), showing HR when attacked by an avirulent gall midge biotype (GMB1), to deduce molecular and biochemical bases of such a complex interaction. Till now, such an integrated analysis of host transcriptome and metabolome has not been reported for any rice-insect interaction.

**Results:**

Transcript and metabolic profiling data revealed more than 7000 differentially expressed genes and 80 differentially accumulated metabolites, respectively, in the resistant host. Microarray data revealed deregulation of carbon (C) and nitrogen (N) metabolism causing a C/N shift; up-regulation of tetrapyrrole synthesis and down-regulation of chlorophyll synthesis and photosynthesis. Integrated results revealed that genes involved in lipid peroxidation (LPO) were up-regulated and a marker metabolite for LPO (azelaic acid) accumulated during HR. This coincided with a greater accumulation of GABA (neurotransmitter and an insect antifeedant) at the feeding site. Validation of microarray results by semi-quantitative RT-PCR revealed temporal variation in gene expression profiles.

**Conclusions:**

The study revealed extensive reprogramming of the transcriptome and metabolome of RP upon GMB1 infestation leading to an HR that was induced by the generation and release of reactive oxygen species i.e. singlet oxygen and resulted in LPO-mediated cell death. RP thus used HR as a means to limit nutrient supply to the feeding maggots and simultaneously accumulated GABA, strategies that could have led to maggot mortality. The integrated results of transcript and metabolic profiling, for the first time, provided insights into an HR+ type of resistance in rice against gall midge.

**Electronic supplementary material:**

The online version of this article (doi:10.1186/s12284-016-0077-6) contains supplementary material, which is available to authorized users.

## Background

The integrated analyses of transcriptomic and metabolomic data obtained from two biological levels i.e. transcript level and metabolite level, respectively, is a useful way of examining the complexity of biological systems. An integrated study of gene expression with metabolomics provides a better insight into the cellular complexity at molecular level. Such an approach was witnessed in the last decade where the emergence of integrated ‘omics’ approach helped us gain valuable insights into the complex route from gene expression (transcriptomics) to function (metabolomics) in living systems under various kinds of stress. Recent literature is replete with examples where data derived from microarray is integrated with gas chromatography–mass spectrometry (GC-MS) based metabolomics data to study various aspects of plant biology such as the differences in root cells and border cells in *Medicago* (Watson et al. 2015), senescence in citrus fruits (Ding et al. 2015), triacylglycerol levels in red alga (Sumiya et al. 2015) and rice response to bacterial blight pathogen (Sana et al. 2010). However, there are only a few reports that describe an integrated transcriptomics and metabolomics approach to study insect-plant interactions and none describing rice-insect interactions.

Plant resistance against invading pests is a complex phenomenon revolving around the success of the plant defense response mounted against the invaders. The incompatible interaction between rice (*Oryza sativa*) and the Asian rice gall midge (AGM, *Orseolia oryzae* Wood-Mason; Diptera, Cecidomyiidae) is such an example of successful host defense that results in maggot mortality which could be accompanied by local necrotic death of host tissue (hypersensitive response, HR+) or without death of host tissue (HR-). The resistance in rice against AGM is governed by a gene-for-gene interaction involving the products of resistance gene (*R*-gene) of the host and avirulence gene (*avr*-gene) of the pest (Nair et al. 2011) and their interaction is shown to be closer to plant-pathogen interaction (Stuart et al. 2012, Rawat et al. 2013). Till now 11 *R*-genes (*Gm1* through *Gm11*), against AGM, have been characterized in the rice germplasm in India (Himabindu et al. 2010) and all of them are dominant *R*-genes except *gm3* and these *R*-genes are being utilized for developing gall midge resistant rice varieties through breeding. However, over-use of resistant rice varieties has led to emergence of new virulent biotypes of AGM (Bentur et al. 2003). Therefore, till date none of the *R*-genes is able to confer resistance against all the seven biotypes of gall midge prevalent in India and thus represents a constant co-evolving mechanism of resistance in rice and virulence in gall midge.

Out of the 11 *R*-genes (against AGM) known in rice, resistance associated with *Gm1* and *Gm8* is without invoking HR while resistance associated with the remaining 9 *R*-genes is manifested with HR (Bentur et al. 2011). Though resistance against gall midge is predominantly manifested with HR and results in maggot mortality, HR is not the prerequisite for maggot mortality (Bentur and Kalode 1996). In a pioneering study (Bentur and Kalode 1996), it was shown that an HR+ *indica* rice variety, Phalguna (harboring *Gm2*), was able to resist a secondary infestation by virulent larva but without the expression of HR following its incompatible interaction with gall midge biotype 1 (GMB1). The current study is based on another HR+ *indica* rice variety RP2068-18-3-5 (referred to as RP henceforth) that shows HR upon attack by gall midge biotype 1 (GMB1). RP harbors *gm3* which confers resistance, manifested with HR, against five of the seven GMBs found in India. Mapped to chromosome 4 of rice, *gm3* is the only recessive *R*-gene identified against gall midge thus far and efforts are underway to clone, characterize and functionally validate this gene (Sama et al. 2014). Thus, the resistance shown by RP against GMB1 is a complex trait which involves expression of HR, inability of the maggots to survive on the resistant host and association with a recessive resistance gene. The interaction between RP and GMB1 has never been studied before and therefore, evaluating the molecular and biochemical changes occurring in the host upon GMB1 attack will help us gain insights into the mechanism of resistance conferred by *gm3*.

Moreover, as resistance against AGM in rice is governed by a single gene understanding the mechanism of resistance will provide us valuable inputs for increasing the durability of each *R*-gene. Till date our understanding of rice-gall midge interaction at the molecular level was based on host transcriptomics involving compatible interaction between *indica* rice varieties and gall midge biotypes i.e. Kavya-GMB4M and TN1-GMB4 and similarly for the incompatible interaction between Kavya-GMB1 and Suraksha-GMB4 (Rawat et al. 2012a,b, 2013). This understanding was furthered by resorting to a metabolomics approach wherein metabolite biomarkers for resistance, susceptibility and infestation were identified during interactions between GMB1 and three different hosts i.e. TN1, Kavya and RP (Agarrwal et al. 2014) but did not provide us a picture of changes occurring in host metabolome at different stages of the interaction. Transcriptomics of the gall midge maggots feeding on susceptible or resistant host provided an insight into the changes occurring in the insect when interacting with its host (Sinha et al. 2012). However, thus far, there has been no integrated approach to study data obtained from the transcriptomics and metabolomics of rice-gall midge interaction. Therefore, in the current study, to understand the resistance mechanism of RP against GMB1 we carried out host transcript and metabolic profiling after GMB1 infestation to investigate the interaction at molecular and biochemical levels. We discuss the results of this study and highlight the complexity of the incompatible interaction, involving HR, and also generate a model based on our current findings and information available in the literature to provide an integrated understanding of resistance in RP against GMB1.

## Results

### Differential expression of genes and accumulation of metabolites in resistant host upon gall midge infestation

Gene expression study was performed on the host (*indica* rice variety RP2068-18-3-5; RP) tissue to evaluate the changes occurring in them upon gall midge infestation during an incompatible interaction with the avirulent biotype (GMB1). The un-infested tissue served as control. In the microarray experiment, the total number of probe sets detected were 57,381 out of which 43,738 probe sets filtered out and passed the quality check after data pre-processing and normalization. A total number of 7598 probes (differentially expressed genes, DEGs) showed statistically significant differential expression between infested (I) and un-infested (UI) host tissues at a *p*-value **≤**0.05. In all, 2861 DEGs were retained in the list after applying a fold change filter (threshold = 2), out of which 1494 were up-regulated in infested tissue while 1367 were down-regulated as compared to un-infested tissues (RP-I/RP-UI; Table 1, Additional file 1: Figure S1). Thus, the number of genes up-regulated upon infestation was marginally higher than the ones down-regulated. Gene Ontology (GO) analysis of the microarray data annotated the DEGs to 285 GO terms. However, after filtering the data at *p*-value **≤**0.05 (corrected p-value) the number of GOs reduced to 249 (Additional file 2: Table S1). Out of these 249 GOs, only those GOs have been plotted which accounted for more than 10 % of total transcripts (Additional file 3: Figure S2). This figure signifies the dominance of biological process followed by metabolic process as GO categories to which the maximum number of transcripts were mapped i.e. more than 80 % and 60 %, respectively, of the total transcripts from the microarray data.

**Table 1:** Summary of microarray results

Number of probes detected	Total number of probes filtered	Total number of DEGs (*p* ≤ 0.05)	Up-regulated^a^ genes	Down regulated^a^ genes
57381	43738	7598	1494	1367

DEGs: Differentially Expressed Genes

^a^Up- or down- regulated genes in rice tissues infested with the Asian rice gall midge biotype 1 (GMB1) as compared to un-infested tissues, at a fold change filter of 2. An *indica* rice variety RP2068-18-3-5 (RP) was used for the current study

The metabolic profiles of the host tissue, at three distinct time points i.e. 24 hai (hours after infestation), 48 hai, and 72 hai, could distinguish RP-I from RP-UI when they were subjected to the Partial Least Squares-Discriminant Analysis (PLS-DA; Additional file 4: Figure S3). Of the total number of metabolites detected in metabolic profiles of host tissues, 45 metabolites showed higher accumulation and 41 metabolites showed lower accumulation in RP-I as compared to RP-UI at one or more time points. Thus, suggesting that a substantial metabolic reprogramming occurred in the host upon gall midge infestation. The metabolites showing differential accumulation (2-fold or more in either direction) in infested tissue as compared to un-infested tissue are discussed below (Additional file 5: Table S2).

### Regulation of carbon metabolism in resistant host upon gall midge infestation

**Fig. 1:**
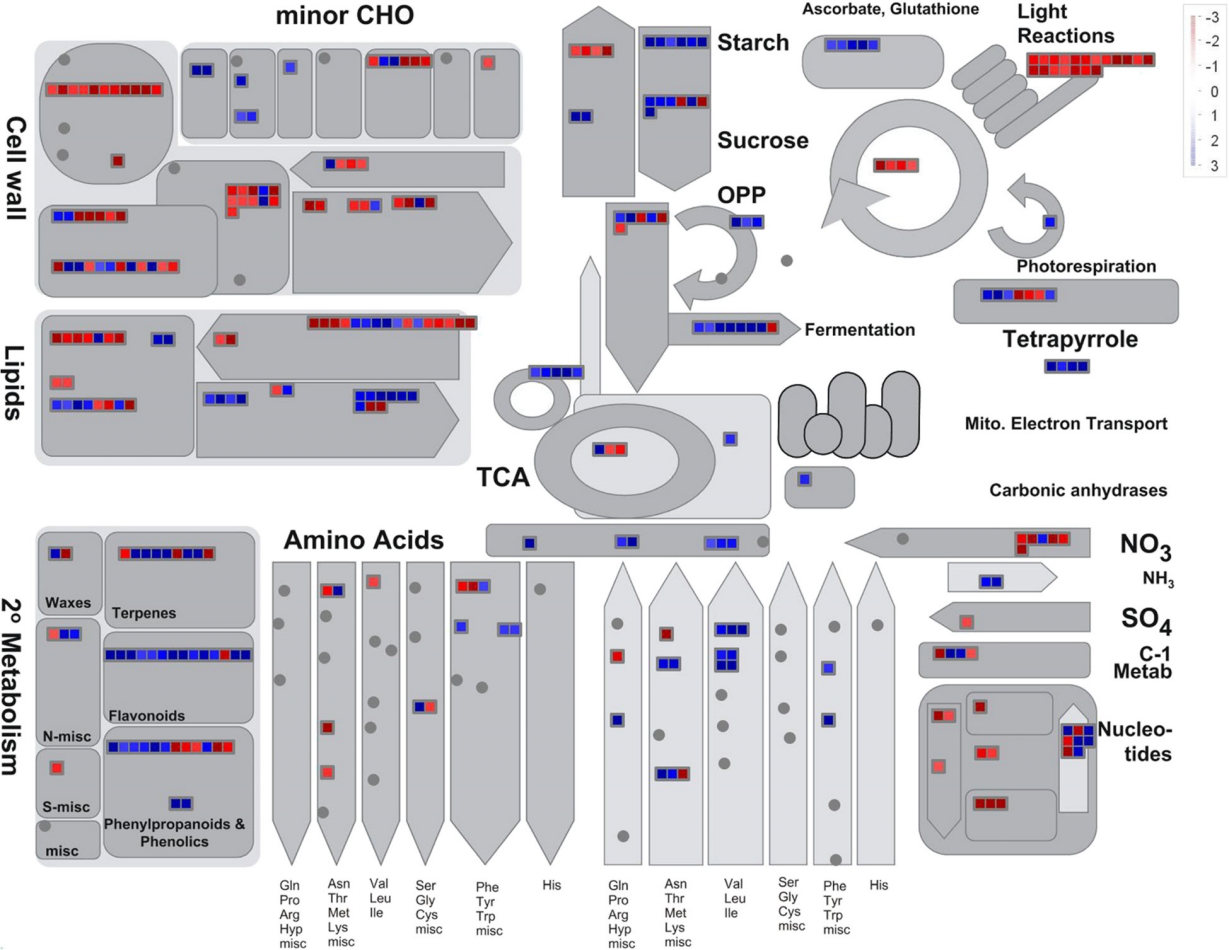
MapMan-based representation of differentially expressed genes (DEGs) onto metabolic pathways. Mapping and distribution of DEGs in *indica* rice variety RP2068-18-3-5 (RP), challenged with the Asian rice gall midge biotype 1 (GMB1), onto metabolic pathway using MapMan software. Transcripts showing more than 2-fold difference between infested (RP-I) and un-infested (RP-UI) tissues of the resistant host RP have been mapped. Transcripts significantly up- and down-regulated are indicated in blue or red, respectively. Grey circles represent genes whose expression did not change more than 2-fold. For detailed information on these transcripts refer Additional file 6: Table S3

**Fig. 2:**
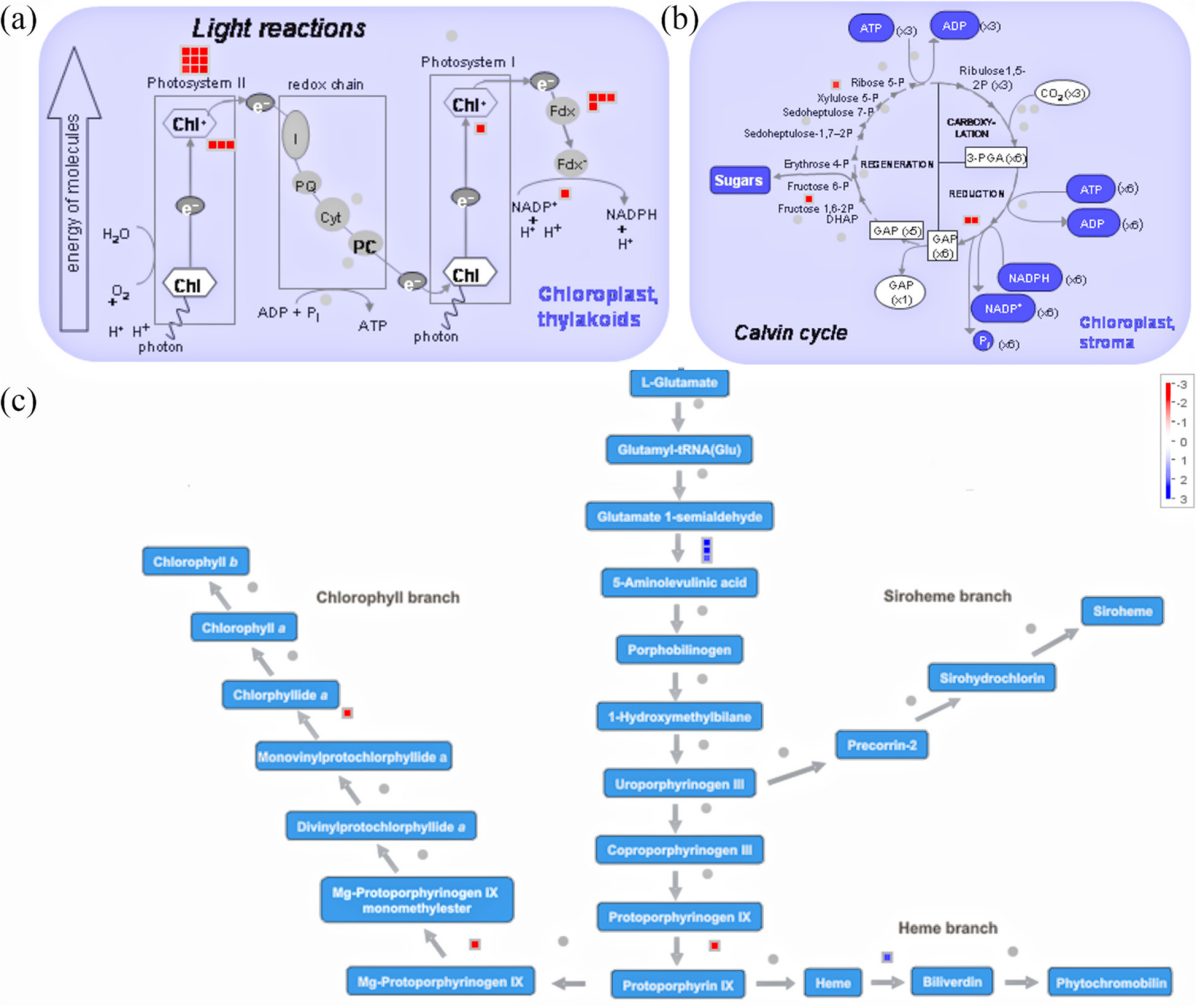
Summary of differentially expressed genes (DEGs) involved in photosynthesis and tetrapyrrole synthesis pathway. Mapping and distribution of DEGs, involved in photosynthesis and tetrapyrrole synthesis pathway (TSP), in *indica* rice variety RP2068-18-3-5 (RP), challenged with the Asian rice gall midge biotype 1 (GMB1). (a) DEGs involved in light reaction of photosynthesis; (b) DEGs involved in Calvin cycle; (c) DEGs involved in TSP. Transcripts showing more than 2-fold change between infested (RP-I) and un-infested (RP-UI) tissues of the resistant host RP have been mapped. Transcripts significantly up- or down-regulated are indicated in blue or red, respectively. Grey circles represent genes whose expression did not change more than 2-fold. For detailed information on these transcripts refer Additional file 7: Table S4

MapMan software (Thimm et al. 2004) was used to map the 2861 DEGs onto several pathways using *Oryza*-specific mapping files. Among the up-regulated pathways were tricarboxylic acid cycle (TCA), oxidative pentose phosphate pathway, fermentation, gluconeogenesis, mitochondrial electron transport and minor and major carbohydrate (CHO) metabolism (Fig. 1, Additional file 6: Table S3). The TCA cycle intermediates such as cis-aconitic acid, 2-oxo-glutaric acid and fumaric acid showed enhanced levels in host tissues upon infestation and so did the transcripts for citrate synthase, the enzyme catalyzing the very first step of this cycle while malic acid showed declined levels. The transcripts involved in Calvin cycle and photosynthesis were down-regulated while those involved in tetrapyrrole synthesis pathway (TSP) were highly up-regulated except a few transcripts, encoding enzymes involved in chlorophyll branch of TSP, which were notably down-regulated (Fig. 2, Additional file 7: Table S4). Of the total transcripts mapped to glycolysis and one-carbon metabolism, 50 % were up-regulated and 50 % were down-regulated (Fig. 1, Additional file 6: Table S3). Pyruvic acid, an important molecule in the glycolytic pathway, showed decline in its levels in infested tissues at 48 hai in accordance with the up-regulation shown by the transcripts encoding pyruvate phosphate dikinase, an enzyme utilizing pyruvate as substrate. Most of the organic acids such as glyceric acid, benzoic acid, malonic acid, adipic acid, oxo-butyric acid, lactic acid and methyl succinic acid involved in various metabolic pathways occurring in plant cells showed a decline in their levels in host tissue upon infestation. The other acids such as carbamic acid, glucuronic acid, threonic acid and hydroxy indole acetic acid showed enhanced accumulation in RP-I as compared to RP-UI. Myristic acid showed an early (at 24 hai) depletion in its levels in infested tissues but at 72 hai its levels went up as high as 17.82 times those found in un-infested tissues. Levels of azelaic acid, a known cellular metabolite in response to stress, were found to be depleted in infested tissues early during incompatible interaction while these levels increased at later stages i.e. 72 hai, the time point which coincides with HR in the host.

#### Differential accumulation of sugars and derivatives

Sugars are the main storage form of photosynthetically fixed carbon in plants. Levels of monosaccharides such as fructose and mannose declined in RP-I tissues as compared to RP-UI. However, at 48 hai, fructose and glucose levels went up in infested tissues as compared to un-infested tissues. The phosphorylated derivatives of hexoses (galactose and glucose) showed enhanced accumulation in infested tissue as compared to un-infested and so did the transcripts encoding for hexokinases and galactokinases. The levels of disaccharide sucrose and turanose declined in infested tissues as compared to un-infested tissues (Additional file 5: Table S2). A corresponding up-regulation of the transcripts encoding invertases, a class of enzyme that catalyzes the breakdown of sucrose to constituting monosaccharides, was observed. Trehalose, another disaccharide, showed elevated levels which was reflected in down-regulation of transcripts involved in trehalose metabolism. The levels of sugar alcohols such as sorbitol, arabinitol and myo-inositol decreased in host tissues upon gall midge challenge which is in line with the up-regulation of transcripts involved in minor CHO metabolism (Fig. 1, Additional file 6: Table S3).

### Regulation of nitrogen metabolism in resistant host upon gall midge infestation

Microarray results revealed that the transcripts involved in polyamine and amino acid metabolism, nitrogen metabolism (especially those encoding for nitrate reductase) were up-regulated (Fig. 1, Additional file 6: Table S3). The analysis of metabolic profiles of host tissues revealed differential accumulation of amino acids and derivatives (Additional file 5: Table S2). Among the amino acids aspartic acid and the un-branched amino acids such as glycine and alanine showed depletion in their levels upon infestation while other amino acids including cysteine, isoleucine, valine, histidine and glutamine showed enhanced accumulation. Levels of leucine, lysine and threonine were elevated which probably triggered the up-regulation of transcripts involved in leucine, lysine and threonine degradation pathway. Aromatic amino acids such as phenylalanine and tyrosine (along with their precursor shikimic acid) and tryptophan, showed an increase in their levels upon infestation which was reflected in the up-regulation of the transcripts involved in the biosynthesis of these aromatic amino acids while the transcripts for shikimate kinase and chorismate synthase were down-regulated upon infestation. Transcripts of simple phenol metabolism were found to be up-regulated which was reflected by the levels of ferulic acid and esculin (tyrosine-derived phenolic acid and coumarin glucoside, respectively) that had depleted in infested tissues as compared to un-infested tissues. The levels of other amino acid precursor/derivative such as acetyl lysine and oxo-proline (or L-pyroglutamate) also went up in infested tissue. The other nitrogenous metabolites such as ornithine and gamma amino butyric acid (GABA) had increased accumulation whereas beta-alanine and urea levels had fallen in infested tissue (Additional file 5: Table S2). In accordance, GABA transaminase, a key enzyme in GABA-shunt was highly up-regulated in the resistant host.

### Regulation of fatty acid and lipid metabolism in resistant host upon gall midge infestation

Transcripts responsible for fatty acid synthesis, fatty acid elongation, fatty acid desaturation, and phospholipid and glycolipid synthesis were also down-regulated upon infestation during incompatible interaction between RP and GMB1 (Additional file 8: Table S5). In addition, transcripts playing a role in lipid degradation and metabolism of ‘exotics’ such as steroids and squalene and xenobiotic degradation were found to be up-regulated (Additional file 8: Table S5). Levels of most of the fatty acids including saturated fatty acids such as arachidic acid, tetradecanoic acid, margaric acid, palmitic acid and stearic acid, and unsaturated fatty acids such as elaidic acid and oleic acid declined in RP-I when compared to RP-UI while docosanoic acid was the only fatty acid to show increased levels in infested host tissues (Additional file 5: Table S2). This was well reflected in down-regulation of transcripts involved in fatty acid synthesis and elongation. Dodecanoic acid was the only fatty acid to show a fluctuation in its levels depending upon the stages of gall midge feeding (Additional file 5: Table S2). Levels of the membrane lipid ethanolamine and its phosphorylated form were enhanced in the infested tissue (Additional file 5: Table S2).

### Regulation of cellular redox state and cell development in resistant host upon gall midge infestation

**Fig. 3:**
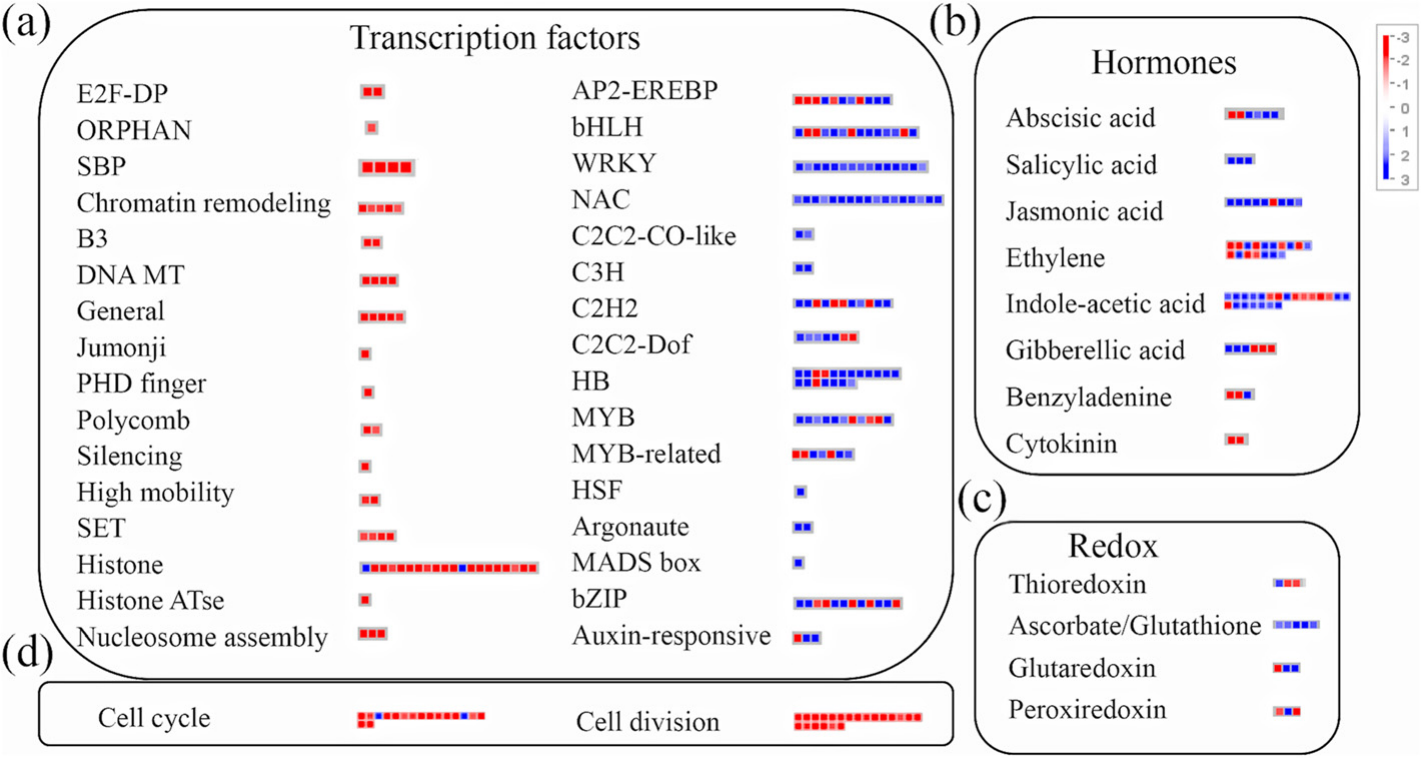
Summary of differentially expressed genes (DEGs) involved in regulation of cellular functioning. Mapping and distribution of DEGs, involved in regulation of cellular functioning, in *indica* rice variety RP2068-18-3-5 (RP), challenged with the Asian rice gall midge biotype 1 (GMB1). (a) DEGs encoding transcription factors; (b) DEGs involved in hormone synthesis; (c) DEGs involved in maintaining cellular redox state; (d) DEGs involved in cellular organization. Transcripts showing more than 2-fold change between infested (RP-I) and un-infested (RP-UI) tissues of the resistant host RP have been mapped. Transcripts significantly up- or down-regulated are indicated in blue or red, respectively. For detailed information on these transcripts refer Additional file 9: Table S6

The transcripts related to redox were up-regulated (Fig. 3). Among the transcripts coding for enzyme families, oxidases were mostly down-regulated while peroxidases were mostly up-regulated (Additional file 8: Table S5). A majority of transcripts involved in cell organization, cell division and cell cycle, cellular vesicle transport as well as storage proteins required during cell development were also found to be down-regulated along with the transcripts involved in cell wall precursor synthesis, cellulose synthesis and cell wall degradation as well as those encoding cell wall proteins including proline rich proteins and pectin esterases. The transcripts representing genes involved in nucleotide metabolism, DNA synthesis and repair, maintenance of chromatin assembly, RNA processing and splicing (including RNA helicases and ribonucleases) were found to be down-regulated (Additional file 8: Table S5).

### Regulation of differential gene expression by transcription factors

Overall the transcription machinery was down-regulated which included several transcription factors such as E2F-DP, ORPHAN, SBP, Chromatin remodelling factors, B3, DNA methyl transferases (MT), general transcription factors, Jumonji, PHD finger, Polycomb group (PcG), silencing group, high mobility group (HMG), SET-domain containing transcription factors. Several transcripts involved in DNA synthesis and repair including histone and histone acetyl transferase, nucleosome/chromatin assembly factors were also down-regulated upon gall midge challenge in RP (Additional file 9: Table S6). Ethylene-responsive element binding protein (EREBP) family, basic helix-loop-helix (bHLH) family, WRKY domain containing family, NAC domain containing family, C3H, C2C2-CO-like, C2H2 and C2C2-Dof zinc finger family, homeobox (HB), MYB-related, MADS box, heat shock factor (HSF), bZIP, Argonaute, auxin and auxin-responsive family of transcription factors were the few up-regulated transcription factors (Fig. 3).

### Semi-quantitative RT-PCR results reveal temporal variation in gene expression in host upon gall midge infestation

**Fig. 4:**
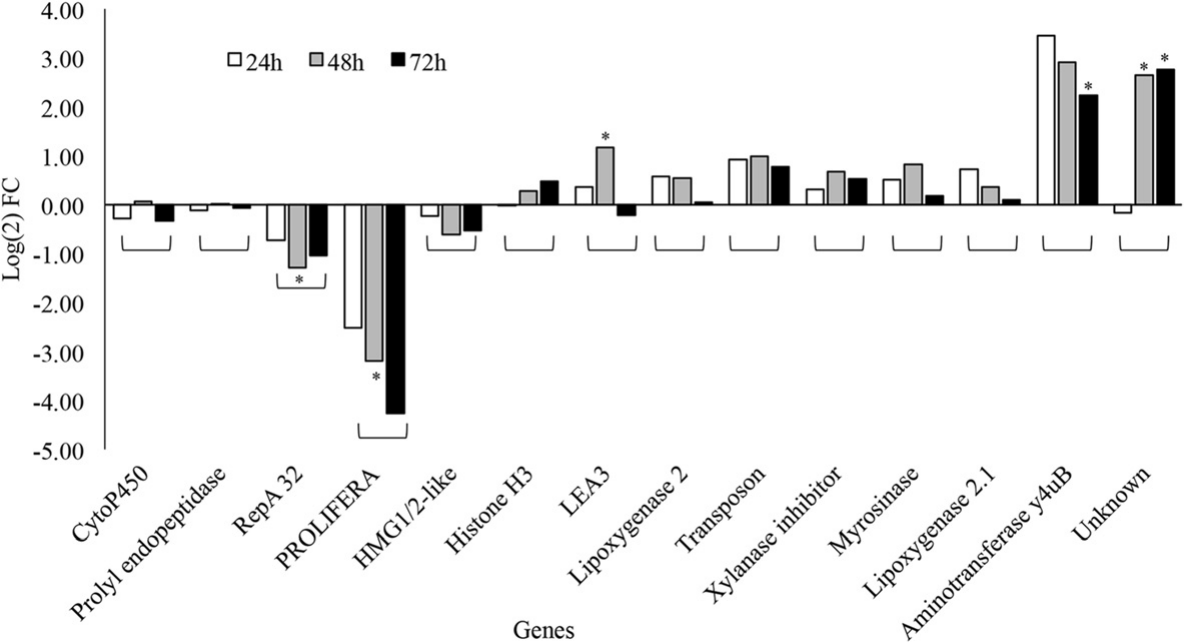
Validation of microarray results by semi-quantitative RT-PCR. Graph represents the expression patterns of genes in *indica* rice variety RP2068-18-3-5 (RP), challenged with the Asian rice gall midge biotype 1 (GMB1), studied at three distinct time points of infestation i.e. 24 h, 48 h and 72 h. Y-axis depicts the log2 values of fold change (FC) in relative expression values (REV) of genes (mentioned on X-axis) between infested (RP-I) and un-infested (RP-UI) samples. Genes with significantly different (*p*-value ≤0.05) REVs between infested and un-infested samples have been indicated by (*) on the bars. RP: *indica* rice variety RP2068-18-3-5; h: hai

Since the microarray results were obtained from pooled RNA samples (see material and methods), the temporal variation in gene expression could not be observed. Therefore, the expression patterns of 14 selected genes showing greater than 10-fold up- or down-regulation were studied further at three time points *viz*., 24, 48, and 72 hai by semi-quantitative RT-PCR (Additional file 10: Table S7). The expression pattern of three genes out of the six most down-regulated genes showed variability at different time points (Fig. 4). Two of these (CytoP450 and prolyl endopeptidase) showed up-regulation at 48 hai while the third gene (Histone H3) showed down-regulation at early hours of infestation (24 hai) but thereafter its relative expression increased in infested tissues as compared to un-infested tissues. Out of the eight most up-regulated genes, two (LEA and an uncharacterized gene) showed down-regulation at 72 hai and 24 hai, respectively (Fig. 4). The expression of aminotransferase y4uB gene was highly up-regulated in RP at all three time points studied. However, when expression patterns were monitored in other rice varieties showing compatible or incompatible interaction (HR-) with GMB1, we did not observe a distinctive expression profile (data not shown).

## Discussion

The over-representation of GO terms such as biological process and metabolic process (Additional file 3: Figure S2 and Additional file 2: Table S1) among the deregulated genes (includes both up- and down-regulated genes) in the host indicates perturbation in the biological and metabolic processes of the resistant host upon gall midge infestation. Concurrent differential accumulation of more than 80 metabolites at one or more time points and the clear distinction in metabolic profiles of infested and un-infested tissues harvested at different time points (as revealed by PLS-DA: Additional file 4: Figure S3), highlights the observed perturbations in the metabolic processes of the resistant host during its interaction with an avirulent gall midge biotype. Our results thus show that extensive reprogramming of the host transcriptome as well as metabolome occurs upon gall midge challenge. This reprogramming of both the biological and metabolic processes could be an attribute of the underlying defense response that is induced in the resistant host upon initiation of insect feeding along with the concomitant recognition of the avirulent gall midge biotype. Furthermore, overlapping results obtained from the microarray and metabolic profiling experiments reveal that host carbon and nitrogen (C/N) metabolism is differentially regulated upon gall midge infestation. The observed decline in levels of carbon-containing compounds and a corresponding increase in nitrogen-containing compounds (Additional file 5: Table S2) indicate a shift in host carbon and nitrogen metabolism (C/N shift) towards N-containing compounds upon gall midge infestation. The coordinated regulation of TCA cycle, glycolysis, minor and major CHO metabolism, amino acid metabolism and nitrogen metabolism (Fig. 1) may have contributed to this C/N shift in the host cells during the incompatible interaction between RP and GMB1. This is in contrast to earlier observations made for the Hessian fly-wheat interaction by Zhu et al. (2008) where a more dramatic C/N shift towards nitrogen in the host was observed during compatible interaction as compared to incompatible interaction with the pest. These observations indicate that C/N metabolism is not only vital to plant growth and development but has a role in plant defense as well. This is further supported by the observation that the ATL31, a C/N metabolism regulator, positively regulates the basal defense response in *Arabidopsis* (Maekawa et al. 2012) by controlling papilla formation during fungal attack (Maekawa et al. 2014). Thus, based on our current findings, we propose that C/N shift is a major reflection of the transcriptomic and metabolomic reprogramming of the host brought about by its interaction with an avirulent pest. In addition, we suggest that though the observed C/N shift may be responsible for the induction of a basal defense in resistant and susceptible hosts alike, this basal defense may be further augmented by other defense responses in an incompatible interaction.

GABA shunt plays a key role in plant C/N metabolism and the growing evidences suggest that it is closely linked to TCA cycle occurring in the cell (reviewed by Fait et al. 2008, Michaeli and Fromm 2015). The three enzymatic reactions occurring in GABA shunt are catalysed by glutamate decarboxylase (GAD), GABA transaminase (GABA-T) and succinic semialdehyde dehydrogenase (SSADH). In our microarray data, the highly up-regulated gene (LOC_Os04g52440; an aminotransferase y4uB), was mapped as GABA-T and was more than 27-fold up-regulated in RP-I as compared to RP-UI (Additional file 6: Table S3), a trend that was validated by semi-quantitative RT-PCR (Fig. 4). The GABA-T mutants of *Arabidopsis* accumulate GABA at much higher levels than wild type (Palanivelu et al. 2003) and are thus resistant to bacterial pathogens (Park et al. 2010). However, in our current study, despite several fold up-regulation of GABA-T transcripts upon infestation, the GABA levels increased in infested tissues of the resistant host (Additional file 5: Table S2), thus suggesting that the observed higher accumulation of GABA in infested tissues is the unutilized substrate after GABA-T activity. Induced accumulation of GABA in pepper plants was suggested to contribute to *R*-gene mediated resistance against *Xanthomonas* and enhanced the cell death mediated defense response in the host against an avirulent pathogen (Kim et al. 2013). Additionally, elevated levels of GABA were shown to contribute towards soybean resistance against leaf roller (Ramputh and Bown 1996) as well as resistance against phytophagous insects (MacGregor et al. 2003) and nematodes (McLean et al. 2003) in transgenic tobacco. Since GABA acts as a neurotransmitter in vertebrates and invertebrates, it is probable that GABA ingested by plant-feeding insects may interfere with normal development of the larva and eventually resulting in larval mortality (Ramputh and Bown 1996). Therefore, we suggest that up-regulation of genes involved in GABA-shunt and induced accumulation of GABA in rice likely play a crucial role in *R*-gene mediated resistance against gall midge.

The resistance shown by RP (conferred by *gm3*) against GMB1 is a complex trait which involves expression of HR and the subsequent inability of the maggots to survive on the resistant host. When attacked by biotrophic pathogens, plants use HR as a strategy to limit the invading pathogen by sacrificing some of their own cells. HR is triggered by oxidative burst within the cells that releases several reactive oxygen species (ROS) such as hydrogen peroxide, superoxide radical and singlet oxygen in the cells under attack. ROS, by-products of aerobic metabolism (Halliwell 2006), are generated in peroxisomes and chloroplasts in plants cells (Foyer and Noctor 2003, Asada 2006). In mammals, ROS production is mostly NADPH-oxidase dependent (Apel and Hirt 2004) while in plants, class III peroxidases may also be responsible for ROS generation. These class III peroxidases have been shown to be effective in mounting a hydrogen peroxide-induced defense response in resistant wheat and in the non-host rice against another Cecidomyiid, the Hessian fly (Liu et al. 2010). While the major ROS scavenging genes were not affected during the Hessian fly-wheat interaction, the genes involved in redox, including the major ROS scavenging enzymes, were mostly up-regulated in the present study (Fig. 3 and Additional file 9: Table S6) and thereby, in all probability, allowing effective scavenging of H_2_O_2_ molecules during rice-gall midge interaction. This indicates that HR observed in RP during its interaction with GMB1 is induced by ROS other than hydrogen peroxide unlike what was observed during the Hessian fly-wheat interaction. It has been shown that the over-expression of ascorbate peroxidase (H_2_O_2_ scavenger) enhanced the intensity of stress response that was mediated by singlet oxygen in *Arabidopsis* (Laloi et al. 2007). Thus, our results point towards the possibility of the presence of an alternate pathway for induction of HR during rice-gall midge interaction that is likely induced by singlet oxygen.

Singlet oxygen-responsive gene networks are, however, not independent of H_2_O_2_ signaling (Murgia et al. 2004) but the interaction between the two reactive species has been described to be antagonistic (Laloi et al. 2007). Singlet oxygen is produced by photosystem II when energy is transferred from excited chlorophyll to oxygen (Krieger-Liszkay 2005) or from molecules acting as potent photosensitizers [protoporphyrin IX, magnesium protoporphyrinogen IX and protochlorophyllide: substrates of the enzymes involved in chlorophyll branch of tetrapyrrole synthesis pathway (TSP)] to oxygen (Meskauskiene et al. 2001, op den Camp et al. 2003). In the current study, the TSP was overall up-regulated in the resistant host upon gall midge attack but, notably, the transcripts coding for enzymes involved in chlorophyll branch of TSP were all down-regulated (Fig. 2). This indicates reduced chlorophyll synthesis that is also reflected by the observed down-regulation of transcripts involved in photosynthesis and Calvin cycle (Fig. 2). This is in line with the reported reduction in photosynthetic activity after initiation of HR in *Arabidopsis* upon *Pseudomonas syringae* infection (Berger et al. 2007). The progressive disintegration of the photosynthetic machinery during HR led to enhanced production of singlet oxygen and singlet oxygen-mediated lipid peroxidation (LPO; Zoeller et al. 2012). Photosynthesis and chlorophyll synthesis are localized in plastids and plastids are present in the dividing apical meristematic cells apart from leaf cells. Rice gall midge maggots feed inside rice plants at the apical meristem, where there is probably little photosynthesis as compared to leaves. But data from our present study clearly showed down-regulation of photosynthesis and chlorophyll branch of TSP and an up-regulation of other transcripts involved in TSP (Fig. 2) in this region of the rice plant after gall midge infestation when compared with un-infested plants. Thus, we suggest that disintegration of photosynthetic machinery leads to generation of singlet oxygen which in turn triggers HR that is observed in the host cells upon gall midge attack.

Under biotic stress, the singlet oxygen-mediated LPO in *Arabidopsis* was further enhanced by enzymatic (lipoxygenases, LOX) and non-enzymatic (free radical induced) peroxidation of membrane lipid leading to accumulation of marker metabolites such as azelaic acid (Triantaphylidès et al. 2008, Zoeller et al. 2012). Though the extent of LPO occurring in host cells was not quantified in the present investigation, azelaic acid levels in infested tissues were found to be lower than that in un-infested tissues at early stages of gall midge feeding (24 hai) but subsequently (72 hai), these levels increased in infested tissues as compared to un-infested tissues (Additional file 5: Table S2). This increase in levels of azelaic acid at later stages of gall midge infestation is indicative of LPO occurring during late stages of infestation when the host cells express HR and die. Additionally, the microarray results revealed several fold up-regulation of transcripts encoding for lipoxygenases (Additional file 8: Table S5) including LOX2 and LOX2.1. Their expression patterns were also confirmed by semi-quantitative RT-PCR (Fig. 4) which showed higher transcript abundance in infested tissues as compared to un-infested tissues at three distinct time points. In accordance with the findings of Zoeller et al. (2012), we suggest that host cell death occurring during HR in RP upon gall midge attack, is due to oxidative damage to cellular membranes triggered by singlet oxygen and which is likely further enhanced by LOX enzymatic activities. Furthermore, microarray results in the current investigation showed several fold up-regulation of transcripts involved in lipid degradation. Previously, it has been shown that mobilization of membrane lipids through lipolysis in resistant wheat plants constituted an important link in its defense against the Hessian fly (Khajuria et al. 2013). Similarly, the oxidation of lipid membranes may in turn help in mediating defense response of rice host against the avirulent gall midge maggots.

To generate a stress response, singlet oxygen signaling is coordinated with other ROS-signaling (Laloi et al. 2007) or phytohormone-signaling (Danon et al. 2005). The involvement of different phytohormones (ethylene, jasmonate, salicylic acid) in modulating the singlet oxygen-mediated cell death during stress in *Arabidopsis* mutants has also been demonstrated earlier (Danon et al. 2005, Ochsenbein et al. 2006). Similarly, a concurrent activation of phytohormone synthesis was observed in the current study which was reflected in up-regulation of genes involved in synthesis of three major phytohormones i.e. ethylene, jasmonate and salicylate (Fig. 3 and Additional file 9: Table S6). Additionally, singlet oxygen has also been shown to control the expression of nuclear encoded genes by means of several transcription factors. In the current investigation, the transcription factors such as ethylene responsive factors (EREBP), WRKY transcription factors, zinc finger proteins, NAC domain containing and MYB were up-regulated in the host after gall midge attack (Fig. 3). Several of these transcription factors have been reported to show quick and enhanced expression in plants upon release of singlet oxygen (Gadjev et al. 2006, Kim et al. 2008) and are known to be involved in plant defense response against biotic stress (van Verk et al. 2009). Thus, results of the current study indicate that the release of singlet oxygen not only triggers HR and lipid peroxidation in the host cells but also controls the host defense against gall midge *via* phytohormones signaling and/or up-regulation of several transcription factors.

The stress response induced by singlet oxygen could be due to its direct cytotoxicity (Meskauskiene et al. 2001) or the singlet oxygen triggered genetic reprogramming of the cells (Wagner et al. 2004). In the current study, many genes involved in DNA synthesis and repair, maintenance of chromatin assembly, cell cycle and cell organization showed several fold down-regulation in RP-I as compared to RP-UI (Fig. 3 and Additional file 9: Table S6) indicating the disorganization of cellular machinery and the preparations of the cells ahead of programmed cell death or HR. The reduced expression of these genes in the resistant host, upon gall midge challenge, was confirmed by semi-quantitative RT-PCR (Fig. 4) and one of them, *Prolifera*, showed statistically significant down-regulation in RP-I as compared to RP-UI. *Prolifera* is a member of mini chromosome maintenance (MCM) protein family involved in DNA replication and was found to be expressed in giant cells in *Arabidopsis* upon successful nematode infection (Huang et al. 2003). Root-knot nematodes alter cell cycle regulation and establish the feeding sites termed as giant cells to draw nutrients from host cells (Goverse et al. 2000). Similarly, genes involved in chromatin remodeling and maintenance were observed to be over-expressed at late stages of giant cell formation in plants during compatible interaction with root-knot nematodes (Portillo et al. 2013). Several genes related to cell organization and DNA synthesis were also found to be up-regulated in the rice host during compatible interactions with gall midge (Rawat et al. 2012b). However, down-regulation of the genes involved in cell organization i.e. *Prolifera*, in the gall midge resistant variety RP during HR, indicates that the avirulent maggots are unable to induce expression of host genes that are involved in establishment/development of feeding site. This may be because, in accordance to plant vigour hypothesis (Price 1991), gall midge (like other galling insects) requires a healthy host for successful establishment of feeding sites and formation of nutritive tissue (Agarrwal et al. 2014) which is not available in the resistant host undergoing cell death, at the maggot feeding site, during HR. Thus, the resistant host is able to prevent the feeding maggots from continued feeding by disrupting the source-sink nutrient channeling by killing some of its own cells at the insect-feeding site during HR.

The pre-dominance of HR in rice gall midge resistance is revealed by the fact that resistance associated with 9 of the 11 *R*-genes known (exceptions *Gm1* and *Gm8*) is manifested with HR (Bentur et al. 2011). However, the HR-induced host cell death at the gall midge feeding site is not the cause of maggot mortality (Bentur and Kalode 1996) and actual death of maggots could be a result of the inability of the maggots to continue feeding on resistant hosts. A recent study on incompatible interaction between another gall midge (*Dasineura marginemtorquens*) and willow (*Salix viminalis*) revealed that the loci for HR and the locus for resistance were not co-located (Höglund et al. 2015). The authors proposed that the resistance of the plant host against the galling insect could be explained by absence of galls i.e. lack of induced susceptibility. Loss-of-susceptibility rather than induced defense has often been used to explain the phenomena of recessive resistance (reviewed by Pavan et al. 2010). However, studies have also shown that recessive resistance is not always associated with loss-of-susceptibility but may also be associated with induced defense response (Gonzalez-Ibeas et al. 2012). Recessive resistance could also mean that the product of the dominant allele acts as a negative regulator of plant immunity and when not expressed in individuals harboring the recessive allele, it leads to the de-repression of the plant defense and consequently resulting in resistance (Pavan et al. 2010). This would probably explain the gall midge resistance phenomenon in RP conferred by *gm3* – the only recessive *R*-gene identified against gall midge thus far.

Thus, the integrated study of rice host transcriptome and metabolome during its interaction with an avirulent biotype of AGM provides important revelations with regard to the resistance mechanism of the host that is associated with a recessive *R*-gene and manifested through HR. Based on our combined analysis of results obtained from transcriptomics and metabolomics, we propose that by expressing HR the host does not kill the invader directly but does so by reducing the nutrient availability to the feeding maggots, by sacrificing some of its own cells, and then hampering the development of the insect by accumulating inhibitory neurotransmitters such as GABA. However, to prove this hypothesis experimentally, an artificial diet for feeding rice gall midge maggots needs to be established. This diet could then be supplemented with antifeedants such as GABA to study their effects on survival rates of maggots. Additionally, the present study revealed novel genes, e.g. the unknown gene (LOC_Os01g40290), that showed maximum fold change in microarray analyses results. These could be evaluated further for understanding their role in conferring resistance against gall midge in particular and also possibly other stresses in general.

## Conclusions

**Fig. 5:**
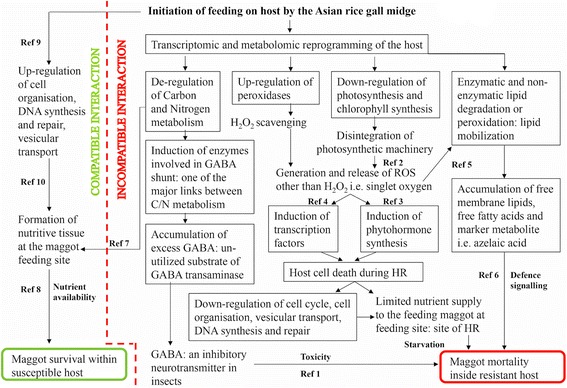
Model representing flow of events occurring during rice-gall midge compatible and incompatible interactions. Representation of flow of events related to rice-gall midge compatible and incompatible interactions (separated by dotted lines). Text within green and red boxes represents outcome of compatible and incompatible interactions, respectively. The attack by the Asian rice gall midge on a resistant host induces extensive transcriptomic and metabolomic reprogramming leading to an array of events that induce hypersensitive response (HR)-mediated host resistance against gall midge. The deregulation of carbon and nitrogen metabolism induces overexpression of genes involved in GABA shunt and accumulation of GABA that could be toxic to the feeding maggots (Ref 1) along with the concomitant release of reactive oxygen species, i.e. singlet oxygen, that trigger HR in the host. The disintegration of photosynthetic machinery leads to generation of singlet oxygen (Ref 2), which further mediates defense signaling involving phytohormones (Ref 3) and transcription factors (Ref 4). Singlet oxygen also induces lipid peroxidation (Ref 5) causing lipid mobilization that plays a role in defense signaling through release of free fatty acids (Ref 6). On the other hand, C/N shift towards nitrogen, during compatible interaction (Ref 7), favors maggot growth by promoting formation of nutritive tissue (Ref 8). Up-regulation of genes involved in cell cycle and organization during compatible interaction (Ref 9) also promotes establishment of nutritive tissue at feeding sites (Ref 10). In contrast, host cellular machinery breaks down at the site of HR during incompatible interaction. Therefore, the host cell death, during HR at feeding site, limits nutrient supply to the feeding maggots and prevents formation of nutritive tissue. The flow of events in the given model is re-constructed based on information available from the current study (text within black boxes) and earlier studies on other insect-plant interactions for which appropriate references have been cited in Additional file 11 : References S1

Based on the results of our study, we conclude that extensive reprogramming of the transcriptome and metabolome occurs in the rice host (RP) upon gall midge (GMB1) infestation leading to an array of events that likely induce hypersensitive response (HR)-mediated host resistance against gall midge. The transcriptomic and metabolic reprogramming lead to a shift in C/N metabolism including induction of enzymes involved in GABA shunt – an important link between C/N metabolism and accumulation of GABA, that could be toxic to the feeding maggots, along with the concomitant release of reactive oxygen species, such as singlet oxygen, that trigger HR in the host. The cell death during HR is likely mediated by lipid peroxidation, releasing free fatty acids which are further involved in defense signaling. The host cell death, as a consequence of HR, and the breakdown of cellular machinery at the feeding site in the resistant host RP do not allow the maggots to continue feeding, leading to their starvation – a demonstration of one of the several defense strategies employed by RP to defend against GMB1 (Fig. 5).

## Methods

### Gall midge infestation and plant sample collection

The Asian rice gall midge (*Orseolia oryzae* Wood-Mason) biotype 1 (GMB1) was used to challenge the resistant rice variety RP2068-18-3-5 (RP henceforth) under greenhouse conditions [at the Indian Institute of Rice Research, (formerly Directorate of Rice Research) Hyderabad] for the current study. The RP seeds were sown in 4 rows, 5 cm apart, with 25 plants in each row, in plastic trays (60 x 30 x 30 cm) that were filled with puddle nutrient enriched soil. After 15 days, plants in some trays were exposed to GMB1 females (*n* = 25) and males (*n* = 10) and plants in other trays were left un-exposed but all trays were treated alike. After 48 h of insect release, all the trays were transferred to humidity chamber (>90 % relative humidity) and were maintained there to facilitate egg hatching and maggot establishment. The plants were periodically checked for maggot establishment and once it was ascertained, this time point was considered 24 hai (hours after infestation). As previously described (Rawat et al. 2010, 2012a) plant tissues (RP-I) were harvested from the feeding site (1 cm region of the meristematic tissue at the maggot feeding site) at 24, 48 and 72 hai. Maggots, both live (at 24 hai and 48 hai) and dead (at 72 hai), were carefully removed from the plant tissues. The tissues from un-infested plants (RP-UI) served as controls and were also excised at the same time points as the infested plants. This exercise was repeated for two consecutive years during *kharif* (monsoon) season (2012 and 2013).

### RNA extraction and microarray for gene expression analysis

Only plant samples (RP-I and RP-UI) from 2013 *kharif* season were used for RNA extraction. The frozen plant tissues (50–100 mg) were ground to a fine powder in liquid nitrogen using a micro-pestle. RNA extraction was carried out using Plant Total RNA Miniprep Kit (mdi Membrane Technologies, USA) as per manufacturer’s instructions. The RNA extracted from tissues representing different time points were pooled and an aliquot of 16 μL (200 ng/μL) was utilized for microarray experiments. Three independent biological replicates were used for transcriptome analyses on GeneChip Rice Genome Array (Affymetrix, USA). The quality of RNA (RIN values) was checked using Agilent Bioanalyzer 2100 (Agilent Technologies, USA). Labeling of RNA, target hybridization, washing-staining-scanning of arrays were carried out by M/s Innovative Life Discoveries (Gurgaon, India). Pre-processing of raw data (.CEL files) was performed using Robust Multichip Average (RMA) algorithm on GeneSpring (Gx13.1) software (Agilent Technologies, USA) and after normalization, only those probe sets having intensities between 10–100 percentile were included in the data. To identify the statistically significant differentially expressed genes (DEGs), moderated *t*-test method (Smyth 2004) was applied and *p*-value cut off 0.05 was considered significant. Only statistically significant DEGs (*p* ≤ 0.05) were subjected to fold change analysis and a filter of 2-fold change between the two conditions i.e. infested versus un-infested was applied and a volcano plot (Cui and Churchill 2003) was constructed (Additional file 1: Figure S1). MapMan software (Thimm et al. 2004) was used to visualize the distribution of DEGs into different bins and their appearance in different metabolic pathways using *Oryza* - specific mapping file. The sequence of events involved in processing of data is presented in Additional file 12: Method S1.

### cDNA synthesis and validation of gene expression results by semi-quantitative RT-PCR

The RNA samples from tissues representing different time points (24, 48 and 72 hai) were used to synthesize the first strand cDNA using Verso cDNA synthesis kit (Thermo Scientific, USA). The protocol was slightly modified and is as follows: the reaction mix containing 1 μL of total RNA along with other constituents of the kit (5X cDNA synthesis buffer, Anchored Oligo dT, dNTP mix, RT enhancer) was incubated at 70 °C for 10 min followed by a quick transfer to 42 °C for another 10 min. Next, the verso enzyme mix was added and the cDNA synthesis was carried out at 42 °C for 90 min after which it was terminated by heating the reaction mix at 95 °C for 2 min. The cDNA, thus synthesized, was cleaned up using QIAquick PCR purification kit (Qiagen, Netherlands) and quantified using NanoVue (GE Healthcare Lifesciences, USA). To validate the expression pattern of highly up-regulated and down-regulated genes, semi-quantitative RT-PCR was performed with cDNA in two technical replicates for each of the three biological replicates. Gene-specific primers for the semi-quantitative RT-PCR were designed using Primer Express Software (Applied Biosystems, USA). The PCR mix (1X; 25 μL) contained 20 ng of cDNA, 0.5 μM primers (forward and reverse each), 200 μM each of the dNTPs, 1.2 Units of Taq polymerase and Taq buffer (Bangalore Genei Pvt Ltd, India). The optimum PCR conditions including cycle number and cDNA amounts were standardized for each gene separately. After the PCR, products were run on 2 % agarose gel (stained with EtBr) at 60 V for 1 h. These gels were then photographed under ultraviolet rays, using the Alpha Imager EP system (Cell Biosciences, USA). The captured images were analyzed using ImageJ software (Schneider et al. 2012). A total of 15 genes were analyzed by semi-quantitative RT-PCR including 8 up-regulated, 6 down - regulated genes and one reference gene. Rice ubiquitin (GenBank accession number NM_001049450; Forward primer- GCGTAGGCTCCTGTTCTTTGG; Reverse primer- AGGGCATCACAATCTTCACAGA) was used as a reference gene for normalization and the fold change values were calculated between the relative expression values (REVs) of infested (RP-I) and un-infested (RP-UI) tissues. A graph was constructed based on the log (2) values of the fold change using MS Excel (Microsoft, USA). The amplification products were cloned and sequenced (M/s Macrogen Inc, South Korea) to confirm their identity.

### Gas chromatography–mass spectrometry (GC-MS) based metabolic profiling

The metabolic profiling was carried out in two independent GC-MS experiments performed on rice tissues, obtained from two consecutive *kharif* season crops, exposed to gall midge under greenhouse conditions. The tissues (RP-I) were exposed to gall midge biotype 1 (GMB1) and plant tissues were excised from stem region at three time points 24, 48 and 72 hai. The stem tissue excised from rice plants not exposed to GMB1 (un-infested, RP-UI) were used as control. The current study involved three independent biological replicates from each year’s sample collection. The infested and un-infested tissues were treated alike and the extracted metabolites after derivatization were analyzed by gas chromatography mass spectrometry (GC-MS). Metabolite extraction and derivatization was performed using the method described earlier (Agarrwal et al. 2014). Briefly, the plant tissue was ground to a fine powder using liquid nitrogen and the metabolites were extracted in cold solvent system comprising of water–methanol-chloroform. The upper water-methanolic phase was dried and derivatized by sequentially using methoxyamine hydrochloride solution in pyridine and N-Methyl-N-(trimethylsilyl)trifluoroacetamide (MSTFA) solution. Similarly, a standard solution containing 18 commonly available compounds was also dried and derivatized (see Agarrwal et al. 2014). The derivatized samples, along with the standard solution, were then analyzed using GC-MS conditions mentioned earlier (Agarrwal et al. 2014). The data was acquired on GCMS 2010QP-PLUS (Shimadzu Corp., Japan) and processed by GCMS solution post run analysis software (Shimadzu Corp., Japan) and peak identification was carried out using NIST 05, NIST 08 and WILEY 08 libraries. The putative identification of metabolites was based on two dimensions i.e. retention index (RI) for GC and similarity index (SI) for MS.

### Statistical analyses of metabolic profiles

The processed data was exported to MS Excel (Microsoft, USA) and the statistical analyses of the metabolic data was carried out using Metaboanalyst (Xia et al. 2015). The fold change in levels of accumulation of various metabolites between the infested and un-infested tissues (RP-I/RP-UI) was calculated followed by a *t*-test. The metabolic data was subjected to multivariate statistical analysis (PLS-DA) which could differentiate the metabolic profiles of infested tissues from those of un-infested tissues at all the three distinct time points.

### Accession numbers for microarray data

The microarray data reported in this article have been deposited in the Gene Expression Omnibus database (http://www.ncbi.nlm.nih.gov/geo/) with accession number GSE70526.

## Additional files

Additional file 1: Figure S1. Volcano plot depicting log2-fold change (x-axis) versus –log10 p-value (y-axis) in gene expression profiles of rice upon GMB1 challenge. RP-I/RP-UI represents the comparison between infested (RP-I) and un-infested (RP-UI) rice tissues. (TIF 1004 kb) Additional file 2: Table S1. Overview of GO analysis. (XLSX 36 kb) Additional file 3: Figure S2. Bar diagram showing results of Gene Ontology (GO) analysis for the distribution of transcripts, identified in rice challenged with the Asian rice gall midge biotype 1 (GMB1), across various biological and cellular processes. Only those GOs accounting for more than 10 % transcripts have been plotted in the graph. (TIF 1757 kb) Additional file 4: Figure S3. Two-dimensional score plots between selected components on the basis of Partial Least Squares–Discriminant Analysis performed on metabolic profiles of infested (RP-I) and un-infested (RP-UI) samples of rice obtained at different time points after gall midge infestation a) 24 h b) 48 h c) 72 h. The X-axis represents components that have maximum variability and y-axis represents components having second highest variability. RP: *indica* rice variety RP2068-18-3-5; h: hai. (TIF 1110 kb) Additional file 5: Table S2. Overview of metabolic profiling results. (DOCX 21 kb) Additional file 6: Table S3. Overview of deregulation of transcripts, involved in metabolism (both primary and secondary metabolism), in the resistant host (RP) upon gall midge biotype 1 (GMB1) infestation. Detailed information such as fold change in relative expression of these transcripts upon gall midge infestation (Fold change RPI/RPUI), their gene locus ids, protein name and function, GO annotation, pathways, Affymetrix probe ids is provided. RP: i*ndica* rice variety RP2068-18-3-5, I: infested, UI: un-infested. (XLSX 73 kb) Additional file 7: Table S4. Overview of deregulation of transcripts, involved in light reaction and Calvin cycle of photosynthesis and tetrapyrrole synthesis pathway, in the resistant host (RP) upon gall midge biotype 1 (GMB1) infestation. Detailed information such as fold change in relative expression of these transcripts upon gall midge infestation (Fold change RPI/RPUI), their gene locus ids, protein name and function, GO annotation, pathways, Affymetrix probe ids is provided. RP: i*ndica* rice variety RP2068-18-3-5, I: infested, UI: un-infested. (XLSX 16 kb) Additional file 8: Table S5. Microarray analyses of rice tissues challenged with the Asian rice gall midge biotype 1 (GMB1). Transcripts have been placed into several bins using MapMan software and information relating to fold change in relative gene expression values (fold change) between infested (RPI) and un-infested tissues (RPUI), Affymetrix gene ids, description of the transcript is provided. RP: *indica* rice variety RP2068-18-3-5. (XLSX 234 kb) Additional file 9: Table S6. Overview of transcripts encoding transcription factors, transcripts involved in hormone synthesis, cell organization and maintaining cellular redox state, in the resistant host (RP) upon gall midge biotype 1 (GMB1) infestation. Detailed information such as fold change in relative expression of these transcripts upon gall midge infestation (RPI/RPUI), their gene locus ids, protein name and function, GO annotation, Affymetrix probe ids is provided. RP: *indica* rice variety RP2068-18-3-5, I: infested, UI: un-infested. (XLSX 57 kb) Additional file 10: Table S7. Details of genes shortlisted, on the basis of microarray results, for semi-quantitative RT-PCR. (DOCX 16 kb) Additional file 11:
**References S1.** List of references referred to in Fig. 5. (DOCX 15 kb) Additional file 12:
**Method S1.** Work flow for microarray analyses. (DOCX 23 kb)

## Abbreviations

CHO: Carbohydrate
DEGs: Differentially expressed genes
GABA: Gamma amino butyric acid
GC-MS: Gas chromatography–mass spectrometry
GMB1: Gall midge biotype 1
GO: Gene ontology
hai: Hours after infestation
HR: Hypersensitive response
LOX: Lipoxygenases
LPO: Lipid peroxidation
PLS-DA: Partial Least Squares-Discriminant Analysis
R-gene: Resistance gene
RIN: RNA integrity number
RMA: Robust Multichip Average
ROS: Reactive oxygen species
RP: *indica* rice variety RP2068-18-3-5
RP-I: RP infested
RP-UI: RP un-infested
RP-I/RP-UI: Fold change difference between RP infested and RP un-infested
RT-PCR: Reverse transcription-polymerase chain reaction
TCA: Tricarboxylic acid cycle
TSP: Tetrapyrrole synthesis pathway
