# Supplementary figures and images for: Metabolic and transcriptomic changes induced in host during hypersensitive response mediated resistance in rice against the Asian rice gall midge
Source: Rice (N Y). 2016 Feb 19;9:5. doi: 10.1186/s12284-016-0077-6 (PMC4759115; doi:10.1186/s12284-016-0077-6)

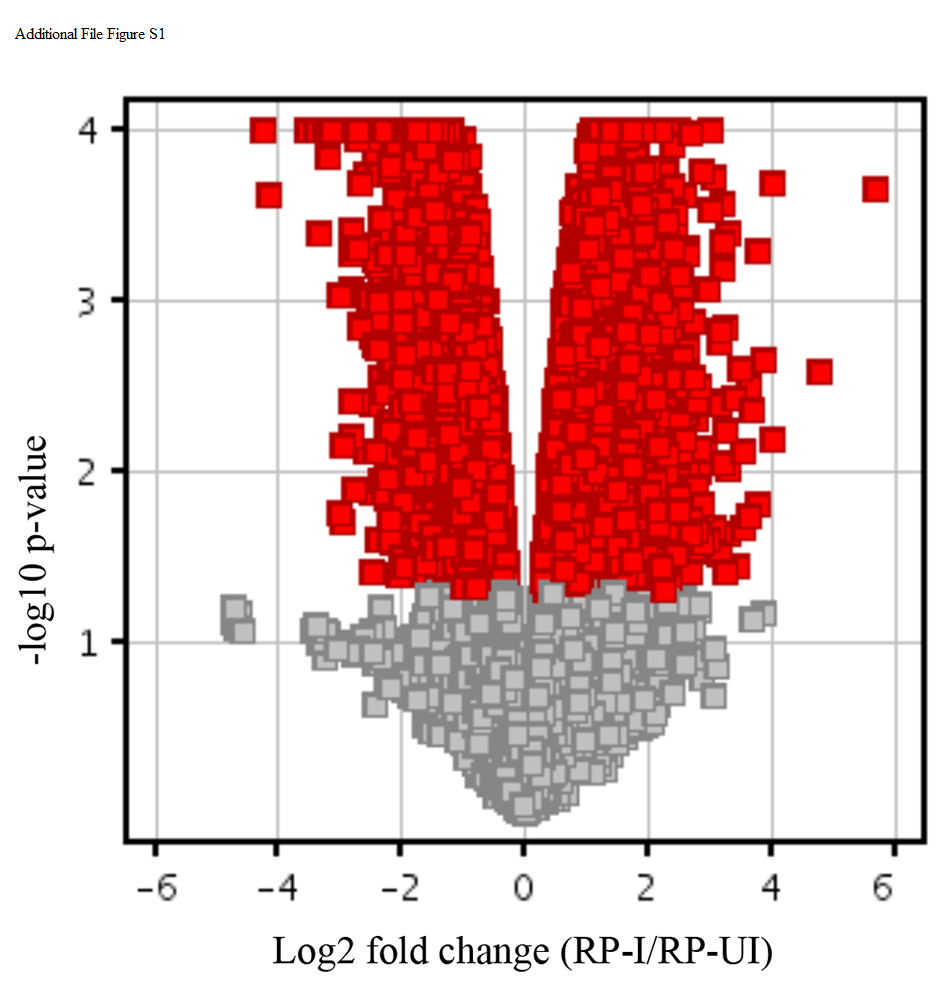

Supplement: Additional file 1: Figure S1. — Volcano plot depicting log2-fold change (x-axis) versus –log10 p-value (y-axis) in gene expression profiles of rice upon GMB1 challenge. RP-I/RP-UI represents the comparison between infested (RP-I) and un-infested (RP-UI) rice tissues. (TIF 1004 kb) [file 12284_2016_77_MOESM1_ESM.tif]

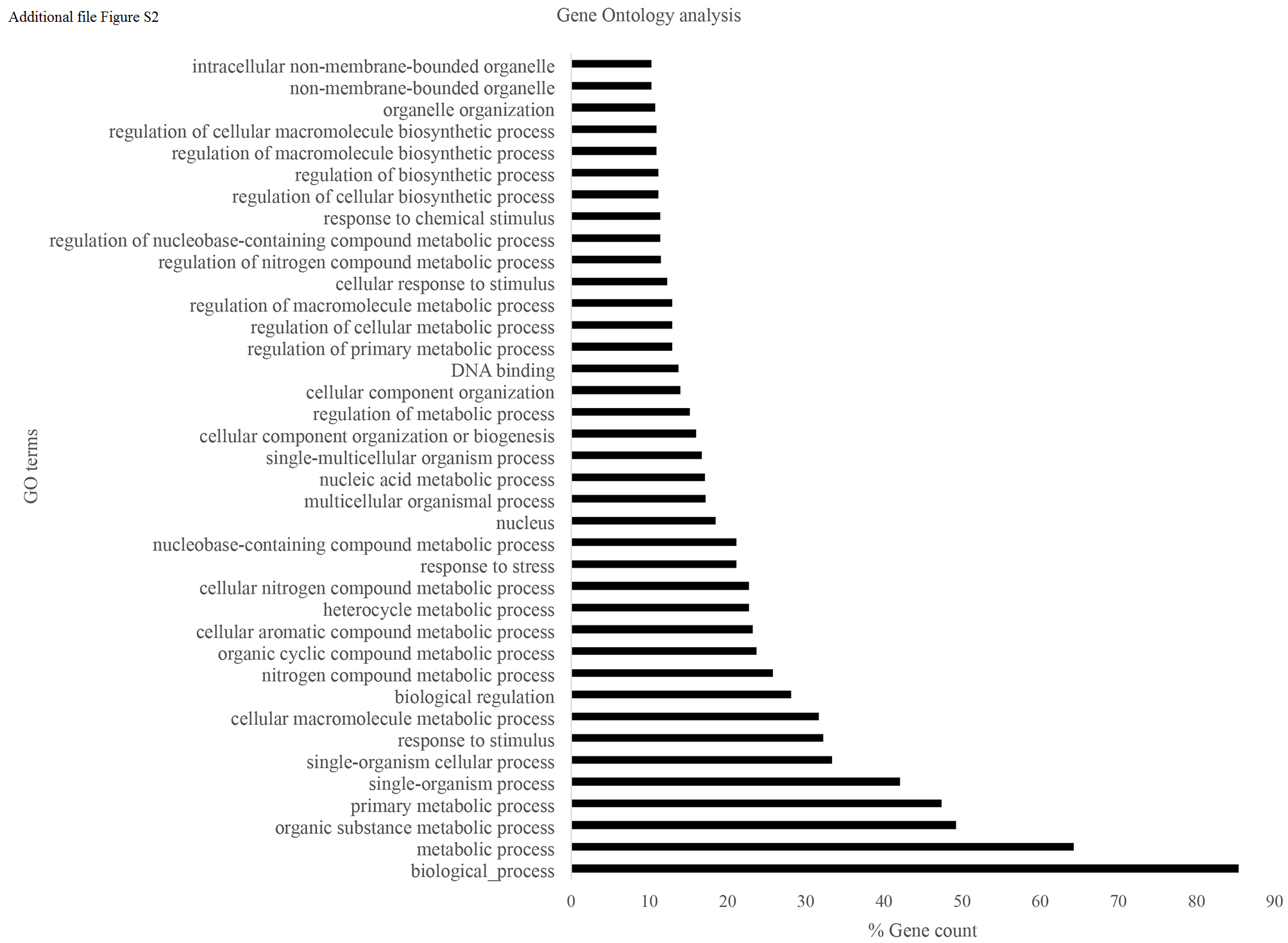

Supplement: Additional file 3: Figure S2. — Bar diagram showing results of Gene Ontology (GO) analysis for the distribution of transcripts, identified in rice challenged with the Asian rice gall midge biotype 1 (GMB1), across various biological and cellular processes. Only those GOs accounting for more than 10 % transcripts have been plotted in the graph. (TIF 1757 kb) [file 12284_2016_77_MOESM3_ESM.tif]

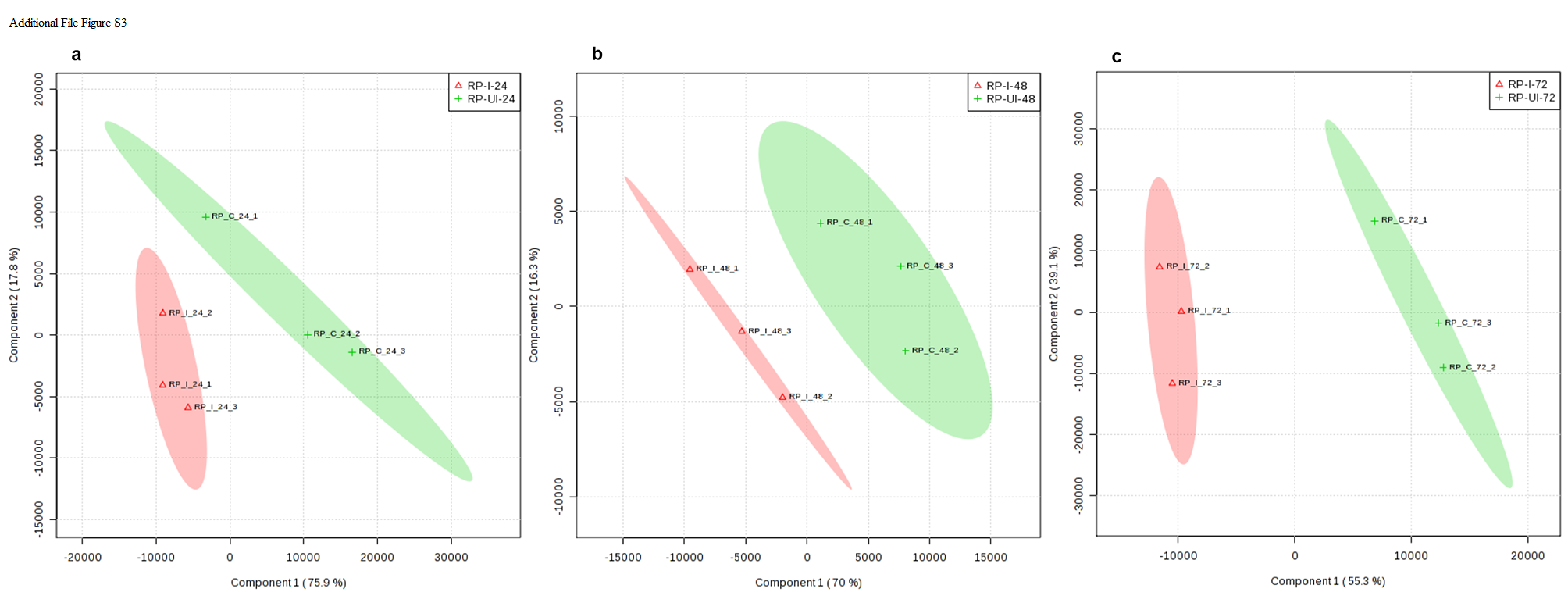

Supplement: Additional file 4: Figure S3. — Two-dimensional score plots between selected components on the basis of Partial Least Squares–Discriminant Analysis performed on metabolic profiles of infested (RP-I) and un-infested (RP-UI) samples of rice obtained at different time points after gall midge infestation a) 24 h b) 48 h c) 72 h. The X-axis represents components that have maximum variability and y-axis represents components having second highest variability. RP: indica rice variety RP2068-18-3-5; h: hai. (TIF 1110 kb) [file 12284_2016_77_MOESM4_ESM.tif]
