# Supplementary material for: Metabolic and transcriptomic changes induced in host during hypersensitive response mediated resistance in rice against the Asian rice gall midge
Source: Rice (N Y). 2016 Feb 19;9:5. doi: 10.1186/s12284-016-0077-6 (PMC4759115; doi:10.1186/s12284-016-0077-6)
Supplement: Additional file 5: Table S2. — Overview of metabolic profiling results. (DOCX 21 kb) [file 12284_2016_77_MOESM5_ESM.docx]

**Additional file Table S2. Distribution of various metabolites across different cellular metabolic pathways and differential accumulation of these metabolites in infested (RP-I) and un-infested (RP-UI) tissues. Data represented here depicts results of two years of data (2012 and 2013 *kharif* season) obtained from metabolic profiles of infested and un-infested samples at three distinct time points i.e. 24, 48 and 72 hai (hours after infestation). RP: *indica* rice variety RP2068-18-3-5**

| BIN NAME | METABOLITE NAME |  | 2012 |  |  | |  |  | 2013 | |
| --- | --- | --- | --- | --- | --- | --- | --- | --- | --- | --- |
|  |  | RP-I/RP-UI_24hai | RP-I/RP-UI_48hai | RP-I/RP-UI_72hai |  | RP-I/RP-UI_24hai | | | RP-I/RP-UI_48hai | RP-I/RP-UI_72hai |
| Minor CHO  Metabolism | Trehalose |  |  |  |  |  | | | 2.43 |  |
|  | Myoinositol | 0.54 |  | **0.38** |  |  | | |  |  |
|  | Myoinositol phosphate | nc |  |  |  |  | | |  |  |
|  | Arabinitol |  |  | 0.04 |  |  | | |  |  |
|  | Turanose | 0.09 | 0.35 | 0.11 |  |  | | |  |  |
|  | Esculin |  | **4.50** | 0.52 |  |  | | |  |  |
|  | Sorbitol | 0.25 |  | **0.30** |  |  | | |  | 0.42 |
| Sugar metabolism | Sucrose | **0.42** |  | **0.25** |  |  | | |  |  |
|  | Fructose | 0.44 | 3.80 | 0.36 |  |  | | |  |  |
|  | Galactose | nc |  |  |  |  | | |  |  |
|  | Galactose-phosphate |  |  |  |  | **2.82** | | |  |  |
|  | Glucose |  | 89.28 | 2.97 |  |  | | |  |  |
|  | Glucose-phosphate |  |  |  |  | **3.35** | | | 2.25 |  |
|  | Hexopyranose |  |  |  |  |  | | |  | 0.43 |
|  | Mannose |  |  |  |  | 0.09 | | |  | 0.41 |
| TCA cycle | Pyruvic acid |  | 0.15 |  |  |  | | |  |  |
|  | Citric acid | nc | **1.91** |  |  |  | | |  |  |
|  | Cis-aconitic acid |  |  |  |  |  | | | **7.92** | 3.04 |
|  | Oxo-glutaric acid | **1.77** | 4.90 |  |  |  | | |  | 5.39 |
|  | Succinic acid | nc |  |  |  |  | | |  |  |
|  | Fumaric acid |  |  |  |  | 2.14 | | |  | 8.86 |
|  | Malic acid |  |  | 0.51 |  |  | | |  |  |
| Photorespiration | Glycerate | 0.45 | **0.06** |  |  | 2.93 | | |  |  |
| Fatty acids | Arachidic acid |  |  |  |  | 0.27 | | |  |  |
|  | Docosanoic acid |  |  |  |  |  | | |  | 2.24 |
|  | Dodecanoic acid |  |  |  |  | 0.55 | | |  | 2.46 |
|  | Elaidic acid |  |  |  |  | 0.33 | | |  |  |
|  | Margaric acid |  |  |  |  | 0.23 | | |  |  |
|  | Oleic acid | 0.54 |  | 0.46 |  | **0.11** | | |  | 18.1 |
|  | Palmitic acid | 0.46 | 0.42 | **0.39** |  | **0.23** | | |  | 3.57 |
|  | Palmitoleic acid | nc |  |  |  |  | | |  |  |
|  | Stearic acid | 0.44 | 0.01 | **0.01** |  | 0.38 | | |  | 2.93 |
|  | Tetradecanoic acid |  | 0.24 |  |  |  | | |  |  |
| Lipid  metabolism | Ethanolamine |  |  |  |  | **2.65** | | |  |  |
|  | Ethanolamine-phosphate |  |  | 2.36 |  |  | | |  |  |
| Miscellaneous organic acids | Benzoic acid | **0.26** | 0.35 | **0.08** |  |  | | |  |  |
|  | Carbamic acid |  | 8.86 |  |  |  | | |  |  |
|  | Glucuronic acid |  |  | 2.98 |  |  | | |  |  |
|  | Oxo-butyric acid |  |  |  |  |  | | | 0.38 |  |
|  | Lactic acid | 0.35 | 0.20 | 0.10 |  | **42.55** | | | 0.53 |  |
|  | Malonic acid | 0.16 |  | 0.32 |  |  | | |  |  |
|  | Methylsuccinic acid |  |  |  |  | 0.42 | | | 0.48 |  |
|  | Adipic acid |  |  |  |  | **0.07** | | | **0.35** |  |
|  | Myristic acid |  |  |  |  | 0.06 | | |  | 17.8 |
|  | Oxalic acid | nc |  |  |  |  | | |  |  |
|  | Threonic acid |  | 2.70 |  |  | 3.17 | | | **2.99** |  |
| Amino acids | Alanine | 0.37 |  | 0.27 |  |  | | | 0.50 |  |
|  | Asparagine | nc |  |  |  |  | | |  |  |
|  | Aspartic acid |  | 0.29 |  |  |  | | |  |  |
|  | Cysteine |  | 2.26 | 6.28 |  |  | | |  |  |
|  | Glutamine |  | 56.21 |  |  |  | | |  | 0.49 |
|  | Glycine | 0.43 | 0.38 | 0.38 |  |  | | |  | 0.47 |
|  | Histidine |  |  | 15.08 |  |  | | |  |  |
|  | Isoleucine |  | 27.37 | **0.37** |  |  | | |  | 3.04 |
|  | Leucine |  |  |  |  |  | | | **4.24** | 17.0 |
|  | Lysine | 2.67 | **4.91** |  |  | 2.14 | | |  |  |
|  | Phenylalanine | **3.49** | 3.23 | 3.89 |  | **3.06** | | |  |  |
|  | Serine | nc |  |  |  |  | | |  |  |
|  | Threonine |  | **2.40** |  |  |  | | |  |  |
|  | Tryptophan | 28.75 | **52.17** | **28.90** |  |  | | |  |  |
|  | Tyrosine | **33.16** | **25.27** | 16.09 |  | **2.74** | | | 0.36 | 0.05 |
|  | Valine |  |  |  |  | 2.15 | | |  |  |
| Amino acid derivatives or precursors | Acetyl lysine |  |  |  |  | 4.06 | | |  |  |
|  | N-formylglycine | nc |  |  |  |  | | |  |  |
|  | Oxoproline |  | **106.72** |  |  |  | | |  |  |
|  | Shikimic acid |  |  |  |  | 3.44 | | |  | 13.5 |
| Nitrogen metabolism | Beta alanine |  | 0.46 |  |  |  | | |  |  |
|  | GABA |  | 2.47 |  |  | **3.93** | | |  |  |
|  | Ornithine |  | **2.39** | 2.38 |  |  | | |  |  |
|  | Urea |  | 0.42 |  |  |  | | |  |  |
| Nucleotide metabolism | Adenine |  |  |  |  | 2.27 | | |  |  |
|  | Deoxyribose | 0.0096 | **0.007** |  |  |  | | |  |  |
|  | Ribose |  | 0.24 | 0.29 |  |  | | | **2.13** | **4.69** |
|  | Inosine |  |  |  |  | 2.20 | | | **1.65** |  |
|  | Uridine |  | 2.80 |  |  |  | | | 5.07 | **5.27** |
|  | Uracil |  |  |  |  | 0.22 | | |  |  |
| Phosphate metabolism | Phosphoric acid | nc |  |  |  |  | | |  |  |
|  | Dimethylphenylphosphonate |  | 2.78 |  |  |  | | |  |  |
|  | Methyl-phosphate | nc |  |  |  |  | | |  |  |
| Phenolic acids | Ferulic acid |  |  |  |  | 0.13 | | |  |  |
|  | Gentisic acid | nc |  |  |  |  | | |  |  |
| Stress-related | Azelaic acid |  |  |  |  | 0.30 | | |  | 3.35 |
| Hormone metabolism | Hydroxy indole acetic acid | 2.46 | 3.59 |  |  |  | | |  |  |
| Redox-related | Irganox |  | 0.44 |  |  |  | | |  |  |

RP-I/RP-UI depicts the difference in levels of metabolites in RP2068-18-3-5 tissues infested with GMB1 as compared to unexposed tissues.

Color shading corresponds to the magnitude of fold change in accumulation of metabolites between RP-I and RP-UI

nc: no change in levels of metabolites between RP-I and RP-UI
