# Supplementary material for: Metabolic and transcriptomic changes induced in host during hypersensitive response mediated resistance in rice against the Asian rice gall midge
Source: Rice (N Y). 2016 Feb 19;9:5. doi: 10.1186/s12284-016-0077-6 (PMC4759115; doi:10.1186/s12284-016-0077-6)
Supplement: Additional file 10: Table S7. — Details of genes shortlisted, on the basis of microarray results, for semi-quantitative RT-PCR. (DOCX 16 kb) [file 12284_2016_77_MOESM10_ESM.docx]

**Additional file Table S7. Details of genes shortlisted (based on microarray results) for semi-quantitative RT-PCR**

| Name | Bin Name | Locus ID | Description | FC (microarray) | Representative Public ID | Primer sequence |
| --- | --- | --- | --- | --- | --- | --- |
| CytoP450 | misc.cytochrome P450 | loc_os03g04530 | 12003.m05981 protein cytochrome P450 86A2, putative, expressed | -18.948 | AK105857.1 | \| F: CCGTGGCCTGGAACTTCG  R: TTGGCACACGGTCGATCTAC \| \| --- \| |
| Prolyl endopeptidase | protein.degradation | loc_os04g47360 | 12004.m09681 protein prolyl endopeptidase, putative, expressed | -18.025 | AK065953.1 | F: TCTCACCCGACTCTGCTGTA  R: ACGACACGAACCCGAGATTC |
| RepA 32 | DNA.repair | loc_os02g58220 | 12002.m10843 protein replication protein A 32 kDa subunit, putative, expressed | -11.763 | AB037145.1 | F: CGTCGAGACAGCCAACGTTA  R: GCAGCGGTTTCAGCAGAATC |
| RepA 32 | protein.aa activation | loc_os02g58220 | 12002.m10843 protein replication protein A 32 kDa subunit, putative, expressed | -11.763 | AB037145.1 | Same as above |
| PROLIFERA | DNA.synthesis/chromatin structure | loc_os12g37400 | 12012.m07525 protein PROLIFERA protein, putative, expressed | -10.668 | AK073411.1 | F: CATCCTCCAAGATGTCGCGA  R: CGTGACCCTCTGCAAGAACT |
| HMG1/2-like | RNA.regulation of transcription. Nucleosome/chromatin assembly factor group | loc_os08g01100 | 12008.m04259 protein HMG1/2-like protein, putative, expressed | -10.31 | AK069900.1 | F: GGAGCACCCTGACAACAAGA  R: CATTGTGGTACTCGGCCTTG |
| Histone H3 | DNA.synthesis/chromatin structure.histone | loc_os04g34240 | 12004.m08518 protein histone H3, putative, expressed | -10.107 | CR282432 | F: CATCGTAAGCCGAGCCGATA  R: TACAAGGTTTCCGGTGTCCAA |
| LEA3 | development.late embryogenesis abundant | loc_os05g46480 | 12005.m08771 protein late embryogenesis abundant protein, group 3, putative, expressed | 10.88 | U57641.1 | F: TGAATGATTTCCCTTTGGGTCTA  R: GCGACGACCACCACTTCATA |
| Lipoxygenase 2 | hormone metabolism. jasmonate.synthesis-degradation.lipoxygenase | loc_os03g52860 | 12003.m10259 protein lipoxygenase 2, putative, expressed | 11.723 | AK073529.1 | F: GCGCCATCATCGTCAAGAA  R: GCGGTTGTAGCGATACTTGGA |
| Transposon | misc.cytochrome P450 | loc_os10g37160 | 12010.m06513 protein transposon protein, putative, unclassified, expressed | 13.671 | AK106424.1 | F: GGGAGTTCACCACCGTCATG  R: ATGAGCGCTTCGACTGTTC |
| Xylanase inhibitor | stress.biotic | loc_os11g47600 | 12011.m08560 protein xylanase inhibitor protein 1 precursor, putative, expressed | 13.808 | AK102505.1 | F: CGCTCTACTACGCCGTGATG  R: GGCCGGATTGAATGTGAGAAT |
| Myrosinase | misc.myrosinases-lectin-jacalin | loc_os01g24710 | 12001.m08937 protein salt stress-induced protein, putative, expressed | 16.331 | D10956.1 | F: ACATACGAGGCTGGAGTCCC  R: GGGAATCAAGGGTGGACGTA |
| Lipoxygenase 2.1 | hormone metabolism. jasmonate.synthesis-degradation.lipoxygenase | loc_os12g37260 | 12012.m07511 protein lipoxygenase 2.1, chloroplast precursor, putative, expressed | 16.408 | BM421235 | F: TGTCGGAGGTCGTCAATGG  R: GGAATCGTCCTGCTTCAAGCT |
| Aminotransferase y4uB | amino acid metabolism.synthesis.central amino acid metabolism.GABA.GABA transaminase | loc_os04g52440 | 12004.m10138 protein aminotransferase y4uB, putative, expressed | 27.181 | AK100259.1 | F: TTGGTGCTGGAGGTGTCATC  R: CCAGCCGTCCAAATCCAGTA |
| Aminotransferase y4uB | tetrapyrrole synthesis.GSA | loc_os04g52440 | 12004.m10138 protein aminotransferase y4uB, putative, expressed | 27.181 | AK100259.1 | Same as above |
| Unknown | Unknown | loc_os01g40290 | 12001.m10301 protein expressed uncharacterized protein | 51.741 | AK107734.1 | F: ACTTGCTAGTCAGGCGATCCA  R: CCACTGGGCTAGCTGAACATC |

F: Forward primer sequence; R: Reverse primer sequence

FC: Fold change in gene expression between infested (I) and un-infested (UI) host tissues RP (*indica* rice variety RP2068-18-3-5)
