# Supplementary material for: Metabolic and transcriptomic changes induced in host during hypersensitive response mediated resistance in rice against the Asian rice gall midge
Source: Rice (N Y). 2016 Feb 19;9:5. doi: 10.1186/s12284-016-0077-6 (PMC4759115; doi:10.1186/s12284-016-0077-6)
Supplement: Additional file 11: — References S1. List of references referred to in Fig. 5. (DOCX 15 kb) [file 12284_2016_77_MOESM11_ESM.docx]

**Additional file Reference 1: List of references cited in Figure 5**

Ref 1: Ramputh and Bown 1996

- Ramputh AI, Bown AW (1996) Rapid γ-aminobutyric acid synthesis and the inhibition of the growth and development of oblique banded leaf-roller larvae. Plant Physiol 111: 1349–1352

Ref 2: Zoeller et al 2012

- Zoeller M, Stingl N, Krischke M, Fekete A, Waller F, Berger S, Mueller MJ (2012) Lipid profiling of the *Arabidopsis* hypersensitive response reveals specific lipid peroxidation and fragmentation processes: biogenesis of pimelic and azelaic acid. Plant Physiol 160: 365–378

Ref 3: Danon et al 2005, Ochsenbein et al 2006

- Danon A, Miersch O, Felix G, Camp RG, Apel K (2005) Concurrent activation of cell death-regulating signalling pathways by singlet oxygen in *Arabidopsis thaliana*. Plant J 41: 68–80
- Ochsenbein C, Przybyla D, Danon A, Landgraf F, Gobel C, Imboden A, Feussner I, Apel K (2006) The role of EDS1 (enhanced disease susceptibility) during singlet oxygen-mediated stress responses of *Arabidopsis*. Plant J 47: 445–456

Ref 4: Gadjev et al 2006, Kim et al 2008

- Gadjev I, Vanderauwera S, Gechev TS, Laloi C, Minkov IN, Shulaev V, Apel K, Inzé D, Mittler R, Van Breusegem F (2006) Transcriptomic footprints disclose specificity of reactive oxygen species signaling in *Arabidopsis*. Plant Physiol 141: 436–445
- Kim C, Meskauskiene R, Apel K, Laloi C (2008) No single way to understand singlet oxygen signalling in plants. EMBO Rep 9: 435–439

Ref 5: Zoeller et al 2012

- Zoeller M, Stingl N, Krischke M, Fekete A, Waller F, Berger S, Mueller MJ (2012) Lipid profiling of the *Arabidopsis* hypersensitive response reveals specific lipid peroxidation and fragmentation processes: biogenesis of pimelic and azelaic acid. Plant Physiol 160: 365–378

Ref 6: Khajuria et al 2013

- Khajuria C, Wang H, Liu X, Wheeler S, Reese JC, El Bouhssini M, Whitworth RJ, Chen M-S (2013) Mobilization of lipids and fortification of cell wall and cuticle are important in host defense against Hessian fly. BMC Genomics 14: 423 doi:10.1186/1471-2164-14-423

Ref 7: Zhu et al 2008

- Zhu L, Liu X, Liu X, Jeannotte R, Reese JC, Harris M, Stuart JJ, Chen M-S (2008) Hessian fly (*Mayetiola destructor*) attack causes a dramatic shift in carbon and nitrogen metabolism in wheat. Mol Plant Microbe Interact 21: 70–78

Ref 8: Harris et al 2006

- Harris MO, Freeman TP, Rohfritsch O, Anderson KG, Payne SA, Moore JA (2006) Virulent Hessian fly (Diptera: Cecidomyiidae) larvae induce a nutritive tissue during compatible interactions with wheat. Ann Entomol Soc Am 99: 305-316

Ref 9: Rawat et al 2012

- Rawat N, Neeraja CN, Nair S, Bentur JS (2012) Differential gene expression in gall midge susceptible rice genotypes revealed by suppressive subtraction hybridization (SSH) cDNA libraries and microarray analysis. Rice 5: 8 doi:10.1186/1939-8433-5-8

Ref 10: Goverse et al 2000

- Goverse A, de Almeida Engler J, Verhees J, van der Krol S, Helder JH, Gheysen G (2000) Cell cycle activation by plant parasitic nematodes. Plant Mol Biol 43: 747-761
