# Supplementary material for: Metabolic and transcriptomic changes induced in host during hypersensitive response mediated resistance in rice against the Asian rice gall midge
Source: Rice (N Y). 2016 Feb 19;9:5. doi: 10.1186/s12284-016-0077-6 (PMC4759115; doi:10.1186/s12284-016-0077-6)
Supplement: Additional file 12: — Method S1. Work flow for microarray analyses. (DOCX 23 kb) [file 12284_2016_77_MOESM12_ESM.docx]

**Additional file Method S1**

**Work flow for microarray analyses**

Total RNA Extraction using Plant Total RNA Miniprep Kit (mdi Membrane) from plant tissue excised at different time points.

RNA (pooled RNA from different time points) quality check using Agilent Bioanalyzer 2100 (Agilent)

Transcriptome analyses on GeneChip Rice Genome Array (Affymetrix)

Labelling of RNA, target hybridization (for 16 h at 60 rpm, at 45 °), washing-staining-scanning (scanning of arrays using 3000 7G and scanner software AGCC)

Raw data collection (.CEL files) and pre-processing of data using Robust Multichip Average (RMA) algorithm

Normalization (Data processing, quality control, baseline transformation)

DEGs (Differentially Expressed Genes) selection

Moderated t-test p-value cut off=0.05 and fold change cut off=2

Mapping of DEGs on metabolic pathways using MapMan software

cDNA synthesis from total RNA extracted from plant tissues at different time points using Verso cDNA synthesis kit (Thermo Scientific)

Semi-quantitative RT-PCR for 14 genes under standardized PCR conditions

Image analysis using ImageJ software

Generation of relative expression values (REVs) of 14 genes using ubiquitin as reference gene. Thereafter, using these REVs, fold change between infested tissue and un-infested tissue was calculated
